# Association Between Pulmonary Function Parameters and Apnea Hypopnea Index in Asthmatic Children Living at High Altitude

**DOI:** 10.1155/carj/6769563

**Published:** 2026-08-18

**Authors:** María Isabel Escamilla, Diego Fernando Severiche-Bueno, Jennifer Benavides, Andrea Parra- Buitrago, Elida Dueñas-Mesa, Mauricio Gonzalez-Garcia

**Affiliations:** ^1^ Department of Sleep Medicine, Fundación Neumológica Colombiana, Bogotá, Colombia; ^2^ Department of Pediatrics, Fundación Cardioinfantil, Bogotá, Colombia, cardioinfantil.org

**Keywords:** asthma, children, obstructive sleep apnea, respiratory function tests

## Abstract

**Introduction:**

Obstructive sleep apnea (OSA) has been associated with poorer asthma outcomes in children, including reduced disease control and a greater risk of exacerbation. However, little is known about the relationship between sleep‐disordered breathing severity and pulmonary function in pediatric patients residing at high altitude.

**Methods:**

We conducted a cross‐sectional study including children aged 3–12 years with physician‐diagnosed asthma who underwent overnight polysomnography at a referral center in Bogotá, Colombia (2640 m above sea level), between 2012 and 2017. Clinical characteristics, spirometric measurements, oscillometry parameters, and polysomnographic variables were collected and analyzed. Associations between pulmonary function indices and apnea–hypopnea measures were evaluated using Spearman correlation analysis.

**Results:**

Sixty‐four children were included, of whom 59.4% had moderate‐to‐severe asthma and 59.4% had uncontrolled disease. In oscillometry, airway resistance at 5 Hz (R5) demonstrated an inverse correlation with AHI before bronchodilator administration (*r* = −0.440, *p* < 0.05) and after bronchodilator administration (*r* = −0.491, *p* < 0.05). Similar inverse correlations were observed between R5 and OAHI. Among spirometric variables, postbronchodilator FEV1 was negatively correlated with OAHI (*r* = −0.329, *p* < 0.05). No significant associations were found between OSA severity and asthma severity, body mass index, or nocturnal oxygen saturation.

**Conclusions:**

Our findings revealed an association between the AHI and VEF1 in asthmatic children residing in a tropical city at high altitude. However, the results regarding the relationship between AHI and R5 were contradictory.

## 1. Introduction

Obstructive sleep apnea (OSA) is one of the most common sleep‐related breathing disorders in childhood and is characterized by recurrent episodes of partial or complete upper airway obstruction during sleep. These events may result in intermittent hypoxemia, sleep fragmentation, and adverse neurocognitive, cardiovascular, and metabolic consequences [1]. Current estimates suggest that OSA affects approximately 2%–5% of children worldwide, although the prevalence may be substantially higher among patients with chronic respiratory diseases including asthma [2, 3].

Asthma is the most prevalent chronic respiratory disorder in children and has been increasingly recognized as an important comorbidity of OSA. Over the last decade, growing evidence has supported a bidirectional relationship between these conditions. Children with asthma are more likely to develop sleep‐disordered breathing, while OSA has been associated with poorer asthma control, increased symptom burden, and a higher frequency of exacerbations [4, 5]. It is well‐established that asthma causes obstructive ventilatory alterations, as evidenced by a decreased forced expiratory volume in one second (FEV1) and an FEV1/CVF ratio below the lower limit of normal, as well as an increase in airway resistance when assessed by oscillometry [6]. With respect to OSA, it can lead to a decrease in functional residual capacity (FRC) and both restrictive and obstructive phenomena [7].

It is known that people living at high altitude have significantly higher forced vital capacity (FVC) and FEV1 parameters than those living at sea level [8, 9]. However, no studies have been conducted in the pediatric population to establish the relationship between asthma and AHI in children using oscillometry and spirometry at high altitude. It is known that approximately 81.6 million people live permanently at high altitude [10–12]. Describing the behavior of asthma and OSA under these geographic conditions is essential to look for differences in diagnostic approach and management and could apply to millions of children living under the same conditions worldwide.

Therefore, the primary objective of this study was to evaluate the association between pulmonary function test results and AHI in asthmatic children aged 3–12 years living in Bogotá, Colombia (2640 meters above sea level). Secondary objectives included describing pulmonary function and polysomnographic characteristics of the cohort, exploring the relationship between oscillometric variables and obstructive apnea–hypopnea index (OAHI) in younger children, assessing the association between asthma severity and OSA severity, comparing nocturnal oxygen saturation across asthma severity categories, and examining the relationship between body mass index (BMI) and sleep apnea severity. Our hypothesis was that the results of pulmonary function tests and AHI would be correlated.

## 2. Materials and Methods

A retrospective cross‐sectional study was performed using data from children with a previous diagnosis of asthma who underwent overnight polysomnography (PSG) at the Fundación Neumológica Colombiana in Bogotá, Colombia, between January 2012 and December 2017. Bogotá is located at 2640 meters above sea level. Eligible participants were between 3 and 12 years of age, a range selected because oscillometry can be reliably performed from early childhood, and the polysomnographic findings are interpreted according to pediatric criteria throughout this age group. The PSG was interpreted according to the American Academy of Sleep rules for children [13].

The statistical program EPIDAT was used to calculate the sample size. We used the previous similar study by Zhang et al, who found an association coefficient of 0.348 between the R5 and the AHI [14]. With a 95% CI and 80% power, the calculated two‐sided sample size was 62 patients.

Children aged 3 to 12 years with a previous physician diagnosis of asthma and at least one available PSG performed at our institution were eligible for inclusion. Participants were excluded if they had other respiratory or systemic conditions that could affect pulmonary function or sleep‐related breathing, including cystic fibrosis, primary ciliary dyskinesia, bronchiolitis obliterans, cardiopulmonary malformations, craniofacial abnormalities, neuromuscular disorders, genetic syndromes, central nervous system diseases, congenital heart defects, or thoracic cage abnormalities. Patients with incomplete clinical records or without signed informed consent from a parent or legal guardian were also excluded. The study protocol was approved by the Ethics Committee of the Fundación Neumológica Colombiana (approval number 201810–23812).

For the purposes of this study, OSA was defined as the presence of an OAHI greater than 1 event per hour of sleep. Disease severity was categorized according to the frequency of obstructive respiratory events as mild (2–5 events/h), moderate (> 5 to < 10 events/h), and severe (≥ 10 events/h). These categories were subsequently used for all comparative and correlation analyses.

Asthma diagnosis was defined according to the parameters established by the Global Initiative for Asthma (GINA) which is based on the diagnosis medical criteria and asthma predictive index (API), and when available PFTs [15]. The severity of asthma was defined according to the degree of GINA control as follows: Mild asthma controlled with inhaled corticosteroid long‐acting‐beta2 adrenergic agonist plus inhaled corticosteroid (ICS‐LABA) as needed, or with low doses of ICS plus long‐acting‐beta2 agonist short (SABA). Moderate asthma: Asthma that is controlled with low or moderate doses of ICS or ICS/LABA. Severe asthma: Asthma requiring high doses of ICS or IC/LABA [15].

### 2.1. Data Collection

Clinical and demographic information was obtained through a detailed review of the patients’ medical records. Data were entered into a standardized electronic collection form specifically developed for the study using Epi Info Version 7.2.2.6. After verification and quality control procedures, the database was exported to Microsoft Excel for storage and validation and subsequently transferred to SPSS Version 24 for statistical analysis. Data extraction was performed by the study investigators, who reviewed the records independently to ensure completeness and consistency of the collected information.

#### 2.1.1. PSG

All participants completed the Pediatric Sleep Questionnaire (PSQ) [16] before undergoing overnight PSG. Sleep studies were performed in a dedicated sleep laboratory using Philips Respironics systems (Alice 5 and Alice LE) and followed the recommendations of the American Academy of Sleep Medicine (AASM) [17]. The recordings included electroencephalography, electrooculography, electromyography, electrocardiography, airflow monitoring, thoracoabdominal respiratory effort, pulse oximetry, body position, snoring detection, and synchronized video monitoring. Children were accompanied by a parent or guardian during the study.

All recordings were manually scored by an experienced sleep technologist and subsequently reviewed by a pediatric pulmonologist trained in sleep medicine. Sleep stages and respiratory events were scored according to the current AASM criteria [17]. The apnea–hypopnea index (AHI) was calculated as the total number of apneas and hypopneas per hour of sleep. Obstructive and central respiratory events were used to derive the OAHI and central apnea–hypopnea index (CAHI), respectively.

Oxygenation parameters included mean oxygen saturation, oxygen desaturation index (ODI), and the percentage of total sleep time spent below oxygen saturation thresholds of 90% (T90) and 85% (T85). Pediatric OSA was defined as an OAHI greater than 1 event per hour of sleep. Severity was classified as mild (2–5 events/h), moderate (> 5 to < 10 events/h), or severe (≥ 10 events/h), and these categories were used in subsequent analyses [17].

#### 2.1.2. Oscillometry

Respiratory impedance was assessed using impulse oscillometry with a Jaeger MasterScreen device. Equipment calibration and quality control procedures were performed according to the manufacturer’s specifications before each testing session. Measurements were obtained during quiet tidal breathing, and respiratory resistance and reactance were analyzed within the 5–20‐Hz frequency range following ATS/ERS recommendations for pulmonary function testing in children [18, 19].

The parameters recorded for analysis included resistance at 5 Hz (R5), resistance at 20 Hz (R20), reactance at 5 Hz (X5), resonant frequency (Fres), the difference between R5 and R20, and the area of reactance (AX). Three technically acceptable maneuvers were obtained from each participant, and only measurements meeting reproducibility and quality criteria were included in the analysis. Following baseline assessment, 400 μg of salbutamol was administered through a pressurized metered‐dose inhaler with a spacer device, and oscillometric measurements were repeated 15 minutes later. Results obtained before and after bronchodilator administration were recorded for subsequent analysis [18, 19].

Tests affected by coughing, swallowing, crying, or other artifacts were excluded. Reproducibility was considered acceptable when the coefficient of variation for resistance measurements was below 10%, and quality control requirements for coherence were fulfilled [18]. Oscillometric values were interpreted using reference equations developed by Gochicoa et al. [20]. Participants were instructed to withhold short‐acting bronchodilators for at least 4 h, long‐acting *β*_2_‐agonists for 12 h, and leukotriene receptor antagonists for 24 h before testing. The use of inhaled corticosteroid therapy was permitted prior to spirometry, if it was not in combination with a LABA.

#### 2.1.3. Spirometry

Spirometric testing was performed before and after bronchodilator administration in accordance with ATS/ERS standards [21]. FVC, FEV1, and the FEV1/FVC ratio were recorded and expressed as percentages of predicted values using the Global Lung Function Initiative (GLI) reference equations [22].

Airflow obstruction was defined by an FEV1/FVC ratio below 80% [23], and the degree of impairment was assessed according to the percentage of predicted FEV1 [24]. Bronchodilator responsiveness was evaluated 20 minutes after administration of 400 μg of inhaled salbutamol. A positive bronchodilator response was defined as an increase greater than 12% in FEV1 and/or FVC compared with baseline values [21].

To minimize the influence of respiratory medications on lung function measurements, participants were instructed to withhold short‐acting bronchodilators for at least 4 h, long‐acting *β*_2_‐agonists for 12 h, and leukotriene receptor antagonists for 24 h before testing. The use of inhaled corticosteroids was permitted when not administered in combination with a long‐acting *β*_2_‐agonist.

### 2.2. Statistical Analysis

Categorical variables were summarized as frequencies and percentages. The distribution of continuous variables was assessed before analysis. Variables with a non‐normal distribution, including age, weight, BMI, AHI, OAHI, CAHI, and pre‐ and postbronchodilator AX values, were reported as medians and interquartile ranges (IQRs), whereas normally distributed variables were described using means and standard deviations.

Comparisons of nocturnal oxygen saturation according to asthma severity were performed using the Mann–Whitney *U* test. Associations between continuous variables, including the relationship between BMI and OAHI, were evaluated using Spearman’s rank correlation coefficient. The relationship between asthma severity and OSA severity categories was examined using the chi‐square test for trend.

All statistical analyses were conducted using SPSS Version 24. A two‐sided *p* value < 0.05 was considered statistically significant.

## 3. Results

A total of 64 children with asthma were included in the study. Of these, 59.4% were male, and the median age was 7 years. The median weight was 23.8 kg, and the median BMI was 16.3 kg/m^2^. No significant correlation was observed between BMI and OAHI (*r* = −0.227; *p* > 0.10). Most participants had moderate‐to‐severe asthma (59.4%), uncontrolled asthma (59.4%), and concomitant allergic rhinitis (92.2%). Tonsillar hypertrophy was absent in 65.6% of the cohort, and alveolar hypoventilation was not identified in the majority of patients (79.7%). Detailed demographic and clinical characteristics are presented in Table 1.

**TABLE 1:** General population characteristics.

Characteristic	*n*	
Age, years	64	7 ± 3 (3–12)
Female	26	26 (40.6)
Male	38	38 (59.4)
Weight, kg	64	23.8 ± 13.57 (11.9–84.4)
BMI, kg/m^2^	64	16.3 ± 4.42 (11–31.1)
Baseline SpO_2_	64	92.5 ± 3 (86–97)
T90	64	1 ± 5.05 (0–95)
AHI	64	2.45 ± 3.65 (0–34.3)
OAHI	64	0.75 ± 1.9 (0–33.1)
CAHI	64	0.35 ± 1.47 (0–5.4)
Controlled asthma	26	26 (40.6)
Mild asthma	26	26 (40.6)
Moderate‐to‐severe asthma	38	38 (59.4)
Allergic rhinitis	64	59 (92.2)
Tonsil hypertrophy	64	22 (34.4)
Alveolar hypoventilation	64	13 (20.3)

*Note:* Results are presented as n (%) for categorical variables and mean ± SD (range) for continuous variables.

Abbreviations: AHI, apnea–hypopnea index; BMI, body mass index; CAHI, central apnea–hypopnea index; OAHI, obstructive apnea–hypopnea index, SpO2, oxygen saturation; T90, percentage of total sleep time with SpO2 < 90%.

**FIGURE 1:**
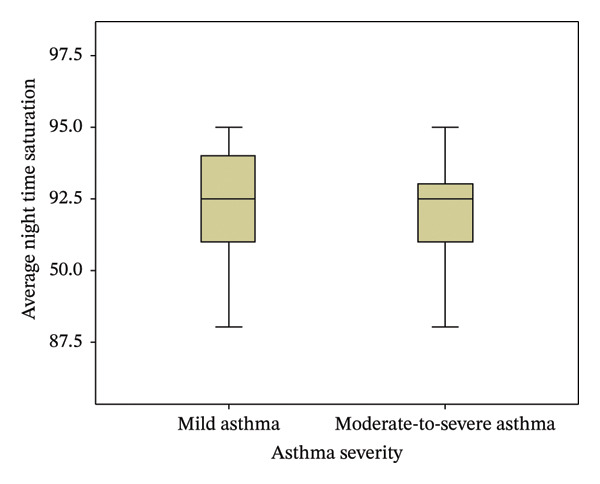
Box plot comparing oxygen saturation by asthma severity.

**FIGURE 2:**
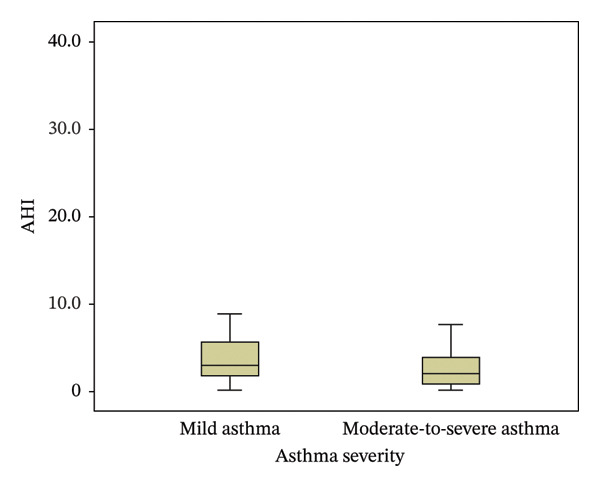
Box plot comparing AHI values by asthma severity.

When participants were stratified according to asthma severity, most children in both the mild and moderate‐to‐severe asthma groups did not meet the criteria for OSA. Similarly, among children classified as having mild, moderate, or severe OSA, the majority belonged to the moderate‐to‐severe asthma category. Although moderate‐to‐severe asthma was more frequently observed among participants with moderate or severe OSA compared with those without OSA, this difference was not statistically significant. The distribution of asthma severity according to OSA classification is shown in Table 2. Mean nocturnal oxygen saturation did not differ significantly between children with mild asthma and those with moderate‐to‐severe asthma (*p* = 0.873). Likewise, no significant association was identified between asthma severity and AHI values (Figures 1 and 2).

**TABLE 2:** Severity of asthma and sleep apnea.

	Mild asthma	Moderate‐to‐severe asthma	Total
No OSA	18 (69.2)	25 (65.8)	43
Mild OSA	5 (45.5)	6 (54.5)	11
Moderate OSA	2 (33.3)	4 (66.7)	6
Severe OSA	1 (25)	3 (75)	4
Total	26	38	64

*Note:* Data are presented as *n* (%).

Pulmonary function test results are summarized in Table 3. In the oscillometric analysis, prebronchodilator R5 values demonstrated a significant inverse correlation with both AHI (*r* = −0.440, *p* < 0.05) and OAHI (*r* = −0.453, *p* < 0.05). Similar findings were observed after bronchodilator administration, with significant negative correlations between R5 and AHI (*r* = −0.491, *p* < 0.05) and between R5 and OAHI (*r* = −0.421, *p* < 0.05). No significant correlations were identified between sleep‐related respiratory parameters and the remaining oscillometric variables, including R20, X5, Fres, R5‐R20, and AX. Regarding spirometric measurements, a negative association was observed between AHI and pulmonary function parameters. Among these, postbronchodilator FEV1 showed a statistically significant inverse correlation with OAHI (*r* = −0.329, *p* < 0.05) (see Table 4).

**TABLE 3:** Pulmonary function test characteristics of the population.

	Variable	
Spirometry	AHI^∗^	1.85 ± 2.93 (0–20.60)
	OAHI^∗^	0.70 ± 1.93 (0–19.10)
	CAHI^∗^	0.25 ± 1.10 (0–4.90)
	Pre‐FEV_1_%^∗∗^	98.6 ± 20.16 (68–151)
	Post‐FEV_1_%^∗∗^	107.74 ± 17.04 (79–145)
	Pre‐FVC%^∗∗^	108.97 ± 14.86 (74–144)
	Post‐FVC%^∗∗^	111.79 ± 11.9 (85–140)
	Pre‐FEV_1_/FVC^∗∗^	86.61 ± 11.28 (62–108)
	Post‐FEV_1_/FVC^∗∗^	94.05 ± 11.14 (69–116)

Oscillometry	AHI^∗^	3 ± 4.93 (0.10–34.30)
	OAHI^∗^	0.95 ± 2.70 (0–33.10)
	CAHI^∗^	0.60 ± 2.18 (0–5.40)
	Pre‐R5%^∗∗^	123.35 ± 28.74 (74–174)
	Post‐R5%^∗∗^	101.08 ± 25.08 (57–158)
	Pre‐R20%^∗∗^	95.92 ± 20.36 (69–141)
	Post‐R20%^∗∗^	85.12 ± 20 (58–126)
	Pre‐AX%^∗^	46.60 ± 135.98 (19.89–21.68)
	Post‐AX%^∗^	29.10 ± 71.45 (10.30–32.12)
	R5‐R20 pre^∗∗^	3.9 (0–1.5)
	R5‐R20 post^∗∗^	2.7 (0–1.2)
	R5‐R20 pre%^∗∗^	32.6 (0–7.3)
	R5‐R20 post%^∗∗^	27.6 (0–8.4)

*Note:* R5, airway resistance at 5 Hz; R20, airway resistance at 20 Hz; AX, area of reactance.

Abbreviations: AHI, apnea–hypopnea index; CAHI, central apnea–hypopnea Index; FVC, forced vital capacity; FEV1, forced expiratory volume in 1 s; OAHI, obstructive apnea–hypopnea index.

^∗^Results are presented as median (IQR).

^∗∗^Results are presented as mean ± SD.

**TABLE 4:** Spearman correlation analysis between pulmonary function tests and sleep‐related respiratory parameters.

	AHI	OAHI	CAHI
Pre‐FEV1 Coefficient (*ρ*) (significance)			
	−0.149 (*p* : 0.373)	−0.301 (*p* : 0.066)	−0.061 (*p* : 0.714)

Pre‐FVC Coefficient (*ρ*) (significance)			
	−0.13 (*p* : 0.438)	0.225 (*p* : 0.174)	−0.004 (*p* : 0.981)

Pre‐FEV1/FVC Coefficient (*ρ*) (significance)			
	−0.078 (*p* : 0.64)	−0.111 (*p* : 0.507)	−0.46 (*p* : 0.004)

Post‐FEV1 Coefficient (*ρ*) (significance)			
	−0.231 (*p* : 0.163)	−0.329 (*p* : 0.044)	−0.144 (*p* : 0.389)

Post‐FVC Coefficient (*ρ*) (significance)			
	−0.184 (*p* : 0.269)	−0.267 (*p* : 0.105)	−0.017 (*p* : 0.92)

Post‐FEV1/FVC Coefficient (*ρ*) (significance)			
	−0.176 (*p* : 0.29)	−0.13 (*p* : 0.435)	−0.57 (*p* : 003)

Pre‐R5 Coefficient (*ρ*) (significance)			
	−0.44 (*p* : 0.024)	−0.453 (*p* : 0.02)	0.317 (*p* : 0.115)

Pre‐R20 Coefficient (*ρ*) (significance)			
	−0.295 (*p* : 0.144)	−0.069 (*p* : 0.739)	−0.024 (*p* : 0.908)

Pre‐R5‐R20 Coefficient (*ρ*) (significance)			
	−0.312 (*p* : 0.129)	−0.302 (*p* : 0.142)	−0.345 (*p* : 0.091)

Pre‐R5‐R20% Coefficient (*ρ*) (significance)			
	−0.091 (*p* : 0.695)	−0.141 (*p* : 0.502)	−0.282 (*p* : 0.172)

Pre‐AX Coefficient (*ρ*) (significance)			
	−0.202 (*p* : 0.323)	−0.207 (*p* : 0.31)	−0.318 (*p* : 0.114)

Post‐R5 Coefficient (*ρ*) (significance)			
	−0.491 (*p* : 0.011)	−0.421 (*p* : 0.032)	0.13 (*p* : 0.527)

Post‐R20 Coefficient (*ρ*) (significance)			
	−0.366 (*p* : 0.066)	−0.123 (*p* : 0.551)	−0.116 (*p* : 0.572)

Post‐R5‐R20 Coefficient (*ρ*) (significance)			
	−0.382 (*p* : 0.060)	−0.329 (*p* : 0.108)	−0.108 (*p* : 0.609)

Post‐R5‐R20% Coefficient (*ρ*) (significance)			
	−0.251 (*p* : 0.227)	−0.256 (*p* : 0.216)	−0.061 (*p* : 0.772)

Post‐AX Coefficient (*ρ*) (significance)			
	−0.387 (*p* : 0.051)	−0.334 (*p* : 0.096)	−0.322 (*p* : 0.11)

*Note:* AX, area of reactance; R5, airway resistance at 5 Hz; R20, airway resistance at 20 Hz.

Abbreviations: AHI, apnea–hypopnea index; CAHI, central apnea–hypopnea index; FEV1, forced expiratory volume in 1 s; FVC, forced vital capacity; OAHI, obstructive apnea–hypopnea index.

## 4. Discussion

The findings of our study indicated a negative association between FEV1 post and OAHI in children residing at high altitudes. This aligns with Wang et al.’s meta‐analysis, which concluded that OSA is associated with lower %FEV1 in children [25]. Similarly, Tsai‐Yu Wang et al. demonstrated in a follow‐up study on the impact of OSA in adults on lung function that there is a greater decline in FEV1 in patients with severe OSA at 5 years vs. mild OSA [26]. Additionally, studies have categorized how lung function is affected by spirometry and oscillometry in the adult population. Van Eyck et al. found an inverse association between FEV1 values and OSA severity, which clearly suggests that the greater the severity of OSA, the lower the lung function [27]. The current evidence suggests that sleep‐disordered breathing is more prevalent in individuals with chronic lung diseases, such as asthma. It is postulated that intermittent hypoxia and oxidative stress may act as a risk factor for the exacerbation of inflammation in both the central and peripheral airways [4, 28].

There is no clear explanation as to why a negative association was found between R5 and AHI. Abdeyrim et al., in a study in an obese adult population, found a significant increase in R5 with AHI [29], contrary to the findings of our study, as well as an inverse relationship between X5 and AHI, suggesting a negative impact of sleep apnea on lung compliance and concluding that an increase in upper airway resistance could have an impact generating greater lung elastance. Rahimi et al. also found an association between R20 and AHI that could be explained by higher central airway resistance associated with higher severity of sleep apnea [30]. In adults [31], the use of R20 has been proposed for the assessment of patients with OSA [32], and the study by Kostrzewska et al. showed that the sensitivity of R5–R20 was higher than the FEV1/FVC ratio in detecting airflow obstruction in patients with OSA [33]; results that were not observed in our study.

The study by Netzer et al. provides a possible explanation. In this study, which was conducted on 6 healthy subjects, they attempted to test the theory that there is a difference in airway resistance between normobaric and hypobaric hypoxia at high altitude. This study documented that there were significant differences between mean expiratory flow and peak flow, proving that lower alveolar and airway pressures due to lower air density result in lower airway resistance [34]. This had already been suggested by Loeppky et al. in 1997 [35] and Richard and Koehle in 2012 [36] and may be one of the explanations for the findings of Dr Finkelstein in 1965 who observed a higher FEV/FVC ratio in COPD patients at high altitude compared to sea level [37, 38]. The rationale behind these findings is that in smaller airways, the gas velocity decreases because the linear velocity is distributed across a larger cross‐sectional area, leading to laminar flow. This is the only type of flow that remains independent of gas density. In contrast, in larger airways, the gas’s linear velocity is higher, and the flow becomes turbulent, which can increase resistance as the air density rises. [39, 40]. In children with asthma, it has been shown that expiratory flow increases when gas density decreases. Therefore, in children living at high altitude, it is possible that although flow limitation may still occur in the larger airways due to turbulent flow, the reduced gas density could lessen the degree of turbulence compared with the conditions at higher barometric pressure, where airflow is more density‐dependent. In the transitional airways, this lower density might even result in Reynolds numbers sufficiently low to favor laminar rather than turbulent flow [39, 41].

It is noteworthy that the resistance of the airways is inversely proportional to the volume of the lungs. At higher lung volumes, attachments from the alveolar walls pull small airways apart, thereby enhancing the effect of pleural pressure on the reduction of airways resistance. In contrast, airway resistance increases significantly during forceful expiration due to the formation of flow‐limiting segments [42]. This could be the reason for the discrepancy between the negative association between R5 and AHI and the negative association between VEF1 and AHI, given that oscillometry is performed with relatively normal breathing, while spirometry is performed with a forced expiration.

It is plausible that an alternative mechanism may be associated with the use of inhaled corticosteroids, which appear to be a risk factor for OSA. This is because the deposition of the molecules in the upper airway can lead to a redistribution of fat at the neck level and generate myopathy of the laryngeal muscles [43, 44]. However, the result between R5 and AHI can also be explained by the use of absolute values, since airway resistance decreases as the airway grows.

Studies in children living at high altitude aged 3 to 5 years [45] and in children aged 7 to 14 years [46] have shown that the normal values of sleep parameters differ from those of children at sea level due to higher total and central AHI and lower saturation. However, it is important to note that the current evidence comes from prospective cross‐sectional studies and some cohorts with small sample sizes that have shown variations between different age groups. Although the study by Ucros et al. sought to determine normal polygraph values in children aged 4–9 years in the city of Chiquinquira at 2560 meters above sea level with a total population of 32 children [47], this study has some limitations such as small sample size and the use of polygraph instead of PSG. These limitations prevent the generalization of its results and the replacement of the criteria established by the AASM. For this reason, we chose to use the values established by the AASM.

This study is innovative in that it is the first to specifically assess differences in lung function in children with asthma with and without OSA using spirometry and oscillometry. Furthermore, it is the first study to be conducted in children with asthma at high altitude, with the findings that may be applicable to nearly 400 million people worldwide who permanently live above 1500 meters.

Some limitations include the absence of a control group residing at low altitude due to the economic and logistical challenges of assembling such a group in our country. Furthermore, the sample size is relatively small, although it was determined based on the study’s primary objective and an association coefficient of 0.3. The limited number of patients with severe OSA, along with the presence of extreme outliers, further restricts the ability to draw conclusions. The total sample size may be insufficient to demonstrate a relationship between OSA and the independent variables that are both clinically significant and statistically significant. Another limitation is the time difference between PSG and pulmonary function tests, which can result in significant variations in pulmonary function within that period. Additionally, the relatively low number of patients with moderate and severe asthma may also have an impact on the oscillometry results. Consequently, we propose to perform a similar study in the future, calculating the sample size in accordance with the minimum difference in proportions. Another important limitation is the potential for selection bias, as only patients who were referred for PSG were included in this retrospective and observational study. Referral for PSG was likely driven by the presence of symptoms or clinical suspicion of sleep‐disordered breathing, which may have increased the probability of identifying OSA within the cohort. Consequently, this could have led to an overestimation of the proportion of OSA among patients with severe asthma and may have strengthened the observed associations. Therefore, the findings may reflect an association with sleep‐disordered breathing in symptomatic individuals rather than with asthma severity itself.

Regarding the control of errors and biases, this study used information available in an institution that provides care as a reference center in a city with particular characteristics of altitude above sea level, climate, and pollution; with this consideration, the extrapolation of the results of the present study is limited in space and time; however, the proposed selection criteria were carefully followed to avoid additional selection biases. Given that the main source of information is secondary, the medical records were reviewed before entering the information into the database, seeking consistency and completeness as a strategy to control information bias. The obtained database was independently reviewed by the researchers in charge of collecting the information, and if there was any doubt about the value, the clinical history was reviewed again.

## 5. Conclusion

Our findings revealed an association between the AHI and VEF1 in asthmatic children residing in a tropical city at high altitude. However, the results regarding the relationship between AHI and R5 were contradictory. Our findings highlight the need for well‐designed studies to evaluate the impact of high altitude on the clinical course of asthma and OSA.

**Nomenclature**
OSA: Obstructive sleep apnea
ULN: Upper limit of normal
BMI: Body mass index
PSG: Polysomnography
SpO2: Oxygen saturation
AHI: Apnea–hypopnea index
OAHI: Obstructive apnea–hypopnea index
CAHI: Central apnea–hypopnea index
FVC: Forced vital capacity
FEV1: Forced expiratory volume in 1 second
R5: Airway resistance at 5Hz
R20: Airway resistance at 20Hz
AX: Area of reactance

## Funding

This study did not receive any funding.

## Disclosure

This manuscript was previously published as a preprint. The preprint is accessible via the following link: https://d197for5662m48.cloudfront.net/documents/publicationstatus/151132/preprint_pdf/b95feb1606102332d37a889d818ca02a.pdf.

Although this article has previously been disclosed as a preprint, it has not undergone peer review in any other journal and has not been accepted for publication elsewhere.

## Conflicts of Interest

The authors declare no conflicts of interest.

## Data Availability

The data that support the findings of this study are available from the corresponding author upon reasonable request.

## References

[1] Abbasi A. , Gupta S. S. , Sabharwal N. et al., A Comprehensive Review of Obstructive Sleep Apnea, Sleep Science. (2021) 14, no. 2, 142–154, 10.5935/1984-0063.20200056. 34381578 PMC8340897

[2] Martin J. , Townshend J. , and Brodlie M. , Diagnosis and Management of Asthma in Children, BMJ Paediatr Open. (2022) 6, no. 1, 10.1136/bmjpo-2021-001277. PMC9045042 35648804

[3] Chang S. J. and Chae K. Y. , Obstructive Sleep Apnea Syndrome in Children: Epidemiology, Pathophysiology, Diagnosis and Sequelae, The Korean Journal of Pediatrics. (2010) 53, no. 10, 863–871, 10.3345/kjp.2010.53.10.863. 21189956 PMC3004499

[4] Garza N. , Witmans M. , Salud M. , Lagera P. G. D. , Co V. A. , and Tablizo M. A. , The Association Between Asthma and OSA in Children, Children. (2022) 9, no. 10, 10.3390/children9101430. PMC9601179 36291366

[5] Porsbjerg C. and Menzies-Gow A. , Co-Morbidities in Severe Asthma: Clinical Impact and Management, Respirology. (2017) 22, no. 4, 651–661, 10.1111/resp.13026. 28328160

[6] Moeller A. , Carlsen K. H. , Sly P. D. et al., Monitoring Asthma in Childhood: Lung Function, Bronchial Responsiveness and Inflammation, European Respiratory Review. (2015) 24, no. 136, 204–215, 10.1183/16000617.00003914. 26028633 PMC9487806

[7] Abdeyrim A. , Zhang Y. , Li N. et al., Impact of Obstructive Sleep Apnea on Lung Volumes and Mechanical Properties of the Respiratory System in Overweight and Obese Individuals, BMC Pulmonary Medicine. (2015) 15, no. 1, 10.1186/s12890-015-0063-6. PMC4513967 26209328

[8] Ortiz-Prado E. , Encalada S. , Mosquera J. et al., A Comparative Analysis of Lung Function and Spirometry Parameters in Genotype-Controlled Natives Living at Low and High Altitude, BMC Pulmonary Medicine. (2022) 22, no. 1, 10.1186/s12890-022-01889-0. PMC8939107 35313848

[9] Lopez Jove O. R. , Arce S. C. , Chavez R. W. et al., Spirometry Reference Values for an Andean high-altitude Population, Respiratory Physiology & Neurobiology. (2018) 247, 133–139, 10.1016/j.resp.2017.09.016. 29017972

[10] Burtscher M. , Effects of Living at Higher Altitudes on Mortality: A Narrative Review, Aging Dis. (2014) 5, no. 4, 274–280, 10.14336/ad.2014.0500274. 25110611 PMC4113517

[11] Luks A. , Ainslie P. N. , Lawley J. S. , Roach R. C. , Simonson T. S. , and Ward M. , Ward, Milledge and West’s High Altitude Medicine and Physiology, 2021, CRC Press, Boca Raton, FL.

[12] Tremblay J. C. and Ainslie P. N. , Global and Country-Level Estimates of Human Population at High Altitude, Proceedings of the National Academy of Sciences of the United States of America. (2021) 118, no. 18, 10.1073/pnas.2102463118. PMC8106311 33903258

[13] Severiche-Bueno D. F. , Escamilla M. I. , Benavides J. , Parra A. , Duenas-Meza E. , and Gonzalez-Garcia M. , Correlation Between Pulmonary Function Tests and the Apnea-Hypopnea Index in Asthmatic Children, Preprint. (2023) 1–9.10.1155/carj/676956342610321

[14] Zhang J. , Zhao J. , Chen M. et al., Airway Resistance and Allergic Sensitization in Children With Obstructive Sleep Apnea Hypopnea Syndrome, Pediatric Pulmonology. (2016) 51, no. 4, 426–430, 10.1002/ppul.23264. 26284311

[15] Venkatesan P. , GINA Report for Asthma, Lancet Respiratory Medicine. (2023) 11, no. 7, 10.1016/s2213-2600(23)00230-8. 37302397

[16] Chervin R. D. , Hedger K. , Dillon J. E. , and Pituch K. J. , Pediatric Sleep Questionnaire (PSQ): Validity and Reliability of Scales for Sleep-Disordered Breathing, Snoring, Sleepiness, and Behavioral Problems, Sleep Medicine. (2000) 1, no. 1, 21–32, 10.1016/s1389-9457(99)00009-x. 10733617

[17] Berry R. B. , Brooks R. , Gamaldo C. et al., AASM Scoring Manual Updates for 2017 (Version 2.4), Journal of Clinical Sleep Medicine. (2017) 13, no. 5, 665–666, 10.5664/jcsm.6576. 28416048 PMC5406946

[18] Beydon N. , Davis S. D. , Lombardi E. et al., An Official American Thoracic Society/European Respiratory Society Statement: Pulmonary Function Testing in Preschool Children, American Journal of Respiratory and Critical Care Medicine. (2007) 175, no. 12, 1304–1345, 10.1164/rccm.200605-642st. 17545458

[19] Oostveen E. , MacLeod D. , Lorino H. et al., The Forced Oscillation Technique in Clinical Practice: Methodology, Recommendations and Future Developments, European Respiratory Journal. (2003) 22, no. 6, 1026–1041, 10.1183/09031936.03.00089403. 14680096

[20] Gochicoa-Rangel L. , Torre-Bouscoulet L. , Martinez-Briseno D. , Rodriguez-Moreno L. , Cantu-Gonzalez G. , and Vargas M. H. , Values of Impulse Oscillometry in Healthy Mexican Children and Adolescents, Respiratory Care. (2015) 60, no. 1, 119–127, 10.4187/respcare.03374. 25336530

[21] Miller M. R. , Hankinson J. , Brusasco V. et al., Standardisation of Spirometry, European Respiratory Journal. (2005) 26, no. 2, 319–338, 10.1183/09031936.05.00034805. 16055882

[22] Quanjer P. H. , Stanojevic S. , Cole T. J. et al., Multi-Ethnic Reference Values for Spirometry for the 3-95-yr Age Range: the Global Lung Function 2012 Equations, European Respiratory Journal. (2012) 40, no. 6, 1324–1343, 10.1183/09031936.00080312. 22743675 PMC3786581

[23] National Asthma E. and Prevention P. , Expert Panel Report 3 (EPR-3): Guidelines for the Diagnosis and Management of Asthma-Summary Report 2007, The Journal of Allergy and Clinical Immunology. (2007) 120, no. 5 Suppl, S94–S138. 17983880 10.1016/j.jaci.2007.09.043

[24] Pellegrino R. , Viegi G. , Brusasco V. et al., Interpretative Strategies for Lung Function Tests, European Respiratory Journal. (2005) 26, no. 5, 948–968, 10.1183/09031936.05.00035205. 16264058

[25] Wang D. , Zhou Y. , Chen R. et al., The Relationship Between Obstructive Sleep Apnea and Asthma Severity and Vice Versa: A Systematic Review and Meta-Analysis, European Journal of Medical Research. (2023) 28, no. 1, 10.1186/s40001-023-01097-4. PMC10062016 36998095

[26] Wang T. Y. , Lo Y. L. , Lin S. M. et al., Obstructive Sleep Apnoea Accelerates FEV(1) Decline in Asthmatic Patients, BMC Pulmonary Medicine. (2017) 17, no. 1, 10.1186/s12890-017-0398-2. PMC5361857 28327130

[27] Van Eyck A. , Van Hoorenbeeck K. , De Winter B. Y. , Van Gaal L. , De Backer W. , and Verhulst S. L. , Sleep-Disordered Breathing and Pulmonary Function in Obese Children and Adolescents, Sleep Medicine. (2014) 15, no. 8, 929–933, 10.1016/j.sleep.2014.03.024. 24985758

[28] Rogers V. E. , Bollinger M. E. , Tulapurkar M. E. et al., Inflammation and Asthma Control in Children with Comorbid Obstructive Sleep Apnea, Pediatric Pulmonology. (2018) 53, no. 9, 1200–1207, 10.1002/ppul.24074. 29862666

[29] Abdeyrim A. , Li N. , Shao L. et al., What Can Impulse Oscillometry and Pulmonary Function Testing Tell Us About Obstructive Sleep Apnea: A Case-Control Observational Study?, Sleep and Breathing. (2016) 20, no. 1, 61–68, 10.1007/s11325-015-1185-z. 25957616

[30] Rahimi B. , Edalatifard M. , Haghighi K. S. , and Kazemzadeh H. , Evaluation of Forced Oscilometry Technique’s Parameters in Severe Obstructive Sleep Apnea Patients Without Breathing Disorder, Journal of Family Medicine and Primary Care. (2020) 9, no. 3, 1492–1496, 10.4103/jfmpc.jfmpc_954_19. PMC7266221 32509639

[31] Liu H. , Ni W. , Zhao J. , Xiong S. , Xu Y. , and Zhang Z. , The Diagnosis Value and Its Implication of Impulse Oscillometry in Obstructive Sleep Apnea Syndrome Patients, Journal of Tongji Medical University. (2000) 20, no. 4, 280–282. 12840911 10.1007/BF02888179

[32] Bednarek M. , Grabicki M. , Piorunek T. , and Batura-Gabryel H. , Current Place of Impulse Oscillometry in the Assessment of Pulmonary Diseases, Respiratory Medicine. (2020) 170, 10.1016/j.rmed.2020.105952. 32843158

[33] Kostrzewska M. , Trafas T. , Brominska B. et al., Airway Obstruction in Sleep Apnea Patients, Advances in Experimental Medicine and Biology. (2019) 1113, 11–17. 29564775 10.1007/5584_2018_161

[34] Netzer N. C. , Rausch L. K. , Friess M. et al., Expiratory Peak Flow and Minute Ventilation are Significantly Increased at High Altitude Versus Simulated Altitude in Normobaria, Life (Basel). (2022) 12, no. 2. 10.3390/life12020306 PMC8875033 35207593

[35] Loeppky J. A. , Icenogle M. , Scotto P. , Robergs R. , Hinghofer-Szalkay H. , and Roach R. C. , Ventilation During Simulated Altitude, Normobaric Hypoxia and Normoxic Hypobaria, Respiration Physiology. (1997) 107, no. 3, 231–239, 10.1016/s0034-5687(97)02523-1. 9128904

[36] Richard N. A. and Koehle M. S. , Differences in Cardio-Ventilatory Responses to Hypobaric and Normobaric Hypoxia: A Review, Aviation, Space and Environmental Medicine. (2012) 83, no. 7, 677–684, 10.3357/asem.3182.2012. 22779311

[37] Finkelstein S. , Tomashefski J. F. , and Shillito F. H. , Pulmonary Mechanics at Altitude in Normal and Obstructive Lung Disease Patients, Aerospace Medicine. (1965) 36, 880–884. 14332342

[38] Luks A. M. and Swenson E. R. , Travel to High Altitude With Pre-Existing Lung Disease, European Respiratory Journal. (2007) 29, no. 4, 770–792, 10.1183/09031936.00052606. 17400877

[39] Macklem P. T. , The Physiology of Small Airways, American Journal of Respiratory and Critical Care Medicine. (1998) 157, no. 5 Pt 2, S181–S183, 10.1164/ajrccm.157.5.rsaa-2. 9606316

[40] Wood L. D. , Engel L. A. , Griffin P. , Despas P. , and Macklem P. T. , Effect of Gas Physical Properties and Flow on Lower Pulmonary Resistance, Journal of Applied Physiology. (1976) 41, no. 2, 234–244, 10.1152/jappl.1976.41.2.234. 956107

[41] Wood L. D. and Bryan A. C. , Effect of Increased Ambient Pressure on Flow-Volume Curve of the Lung, Journal of Applied Physiology. (1969) 27, no. 1, 4–8, 10.1152/jappl.1969.27.1.4. 5786969

[42] Lutfi M. F. , The Physiological Basis and Clinical Significance of Lung Volume Measurements, Multidiscip Respir Med. (2017) 12, no. 1, 10.1186/s40248-017-0084-5. PMC5299792 28194273

[43] Henao M. P. , Kraschnewski J. L. , Bolton M. D. , Ishmael F. , and Craig T. , Effects of Inhaled Corticosteroids and Particle Size on Risk of Obstructive Sleep Apnea: A Large Retrospective Cohort Study, International Journal of Environmental Research and Public Health. (2020) 17, no. 19, 10.3390/ijerph17197287. PMC7579456 33036169

[44] Nguyen-Hoang Y. , Nguyen-Thi-Dieu T. , and Duong-Quy S. , Study of the Clinical and Functional Characteristics of Asthmatic Children with Obstructive Sleep Apnea, Journal of Asthma and Allergy. (2017) 10, 285–292, 10.2147/jaa.s147005. 29066920 PMC5644536

[45] Burg C. J. , Montgomery-Downs H. E. , Mettler P. , Gozal D. , and Halbower A. C. , Respiratory and Polysomnographic Values in 3- to 5-Year-old Normal Children at Higher Altitude, Sleep. (2013) 36, no. 11, 1707–1714, 10.5665/sleep.3134. 24179305 PMC3792389

[46] Grimm M. , Seglias A. , Ziegler L. et al., Sleep Apnea in School-Age Children Living at High Altitude, Pulmonology. (2023) 29, no. 5, 385–391, 10.1016/j.pulmoe.2023.02.008. 36964122

[47] Ucros S. , Granados C. , Hill C. , Castro-Rodriguez J. A. , and Ospina J. C. , Normal Values for Respiratory Sleep Polygraphy in Children Aged 4 to 9 Years at 2560 m Above Sea Level, Journal of Sleep Research. (2021) 30, no. 5, 10.1111/jsr.13341. 33723892

